# Association of Demographic and Early-Life Socioeconomic Factors by Birth Cohort With Dementia Incidence Among US Adults Born Between 1893 and 1949

**DOI:** 10.1001/jamanetworkopen.2020.11094

**Published:** 2020-07-27

**Authors:** Sarah E. Tom, Manali Phadke, Rebecca A. Hubbard, Paul K. Crane, Yaakov Stern, Eric B. Larson

**Affiliations:** 1 Department of Neurology, Columbia University, New York, New York; 2 Department of Epidemiology, Columbia University, New York, New York; 3 Department of Biostatistics, Columbia University, New York, New York; 4 Department of Biostatistics, Epidemiology, and Informatics, University of Pennsylvania, Philadelphia; 5 Department of Medicine, University of Washington, Seattle; 6 Kaiser Permanente Washington Health Research Institute, Seattle

## Abstract

**Importance:**

Early-life factors may be important for later dementia risk. The association between a more advantaged early-life environment, as reflected through an individual’s height and socioeconomic status indicators, and decreases in dementia incidence by birth cohort is unknown.

**Objectives:**

To examine the association of birth cohort and early-life environment with dementia incidence among participants in the Adult Changes in Thought study from 1994 to 2015.

**Design, Setting, and Participants:**

This prospective cohort study included 4277 participants from the Adult Changes in Thought study, an ongoing longitudinal population-based study of incident dementia in a random sample of adults 65 years and older who were born between 1893 and 1949 and are members of Kaiser Permanente Washington in the Seattle region. Participants in the present analysis were followed up from 1994 to 2015. At enrollment, all participants were dementia-free and completed a baseline evaluation. Subsequent study visits were held every 2 years until a diagnosis of dementia, death, or withdrawal from the study. Participants were categorized by birth period (defined by historically meaningful events) into 5 cohorts: pre–World War I (1893-1913), World War I and Spanish influenza (1914-1920), pre–Great Depression (1921-1928), Great Depression (1929-1939), and World War II and postwar (1940-1949). Participants’ height, educational level, childhood financial stability, and childhood household density were examined as indicators of early-life environment, and later-life vascular risk factors for dementia were assessed. Cox proportional hazards regression models, adjusted for competing survival risk, were used to analyze data. Data were analyzed from June 1, 2018, to April 29, 2020.

**Main Outcomes and Measures:**

Participants completed the Cognitive Abilities Screening Instrument every 2 years to assess global cognition. Those with scores indicative of cognitive impairment completed an evaluation for dementia, with dementia diagnoses determined during consensus conferences using criteria from the *Diagnostic and Statistical Manual of Mental Disorders*, 4th edition.

**Results:**

Among 4277 participants, the mean (SD) age was 74.5 (6.4) years, and 2519 participants (58.9%) were women. The median follow-up was 8 years (interquartile range, 4-12 years), with 730 participants developing dementia over 24 378 person-years. The age-specific dementia incidence was lower for those born in 1929 and later compared with those born earlier. Compared with participants born in the pre–Great Depression years (1921-1928), the age- and sex-adjusted hazard ratio was 0.67 (95% CI, 0.53-0.85) for those born in the Great Depression period (1929-1939) and 0.62 (95% CI, 0.29-1.31) for those born in the World War II and postwar period (1940-1949). Although indicators of a more advantaged early-life environment and higher educational level (college or higher) were associated with a lower incidence of dementia, these variables did not explain the association between birth cohort and dementia incidence, which remained when vascular risk factors were included and were similar by sex.

**Conclusions and Relevance:**

Age-specific dementia incidence was lower in participants born after the mid-1920s compared with those born earlier. In this population, the decrease in dementia incidence may reflect societal-level changes or individual differences over the life course rather than early-life environment, as reflected through recalled childhood socioeconomic status and measured height, educational level, and later-life vascular risk.

## Key Points

**Question:**

Are dementia incidence trends by birth cohort associated with early-life environment?

**Findings:**

In this cohort study of 4277 participants in the Adult Changes in Thought study who were born between 1893 and 1949 and were followed up for up to 20 years (1994-2015), the age- and sex-adjusted dementia incidence was lower among those born during the Great Depression (1929-1939) and the period during World War II and postwar (1940-1949) compared with those born in the period before the Great Depression (1921-1928). The association between birth cohort and dementia incidence remained when accounting for early-life socioeconomic environment, educational level, and late-life vascular risk factors.

**Meaning:**

The study’s findings indicate that dementia incidence is lower for individuals born after the mid-1920s compared with those born earlier, and this lower incidence is not associated with early-life environment as measured in this study.

## Teaser

This cohort study examines the association of demographic characteristics and early-life sociodemographic factors by birth cohort with the incidence of dementia among US participants in the Adult Changes in Thought study who were born between 1893 and 1949 and followed up from 1994 to 2015.

## Introduction

Studies have found a stabilization or decrease in dementia incidence in the US and Europe beginning in the 1990s.^1,2,3,4,5^ Although low educational level, midlife hypertension, midlife obesity, hearing loss, late-life depression, diabetes, physical inactivity, smoking, and social isolation have been identified as risk factors for dementia,^6^ previous studies have specifically examined higher educational levels and lower prevalence of midlife vascular risk factors in association with secular trends. Confounding these chronological trends are birth cohort trends, given the major social, economic, and political changes and medical advances that have been associated with increases in standard of living across the life course during the 20th century.^7,8^ The few studies that have assessed dementia incidence trends by birth cohort have found decreasing dementia incidence beginning with cohorts born in the mid-1920s.^9,10,11^

Improvements during the first half of the 20th century with regard to childhood standard of living,^12^ especially improvements in nutrition,^13^ may have implications for dementia risk. The brain develops most rapidly and is most plastic in the first 5 years of life.^14,15^ Strong early brain development supports more complex subsequent neuritic and intraneuronal connections and cognition, conferring lifelong advantage^16^ that may be associated with cognitive reserve.^17^ Previous studies of secular and birth cohort trends in dementia incidence have not considered indicators of neurocognitive development that occurred before education completion.

Using a population-based cohort with up to 20 years of follow-up, we investigated the associations of birth cohort and early-life environment with dementia incidence. We tested the hypotheses that cohorts born more recently would have a lower dementia incidence and that this trend would be associated with higher early-life socioeconomic status and taller height.

## Methods

The Adult Changes in Thought (ACT) study is an ongoing population-based cohort study of incident dementia in a random sample of adults 65 years and older who were born between 1893 and 1949 and are members of Kaiser Permanente Washington (formerly Group Health) in the Seattle region. The institutional review boards of Kaiser Permanente Washington, Columbia University, and the University of Washington approved this study. Written informed consent was obtained from all ACT study participants. This study followed the Strengthening the Reporting of Observational Studies in Epidemiology (STROBE) reporting guideline for cohort studies.^18^

Between 1994 and 1996, 2581 people were enrolled in the ACT study, and between 2000 and 2002, an expansion cohort of 811 people was enrolled. In 2005, the study began ongoing enrollment to replace participants who died, developed dementia, or withdrew from the study, enrolling 120 to 180 participants annually. At study enrollment, participants were dementia-free and completed a baseline evaluation, with subsequent study visits every 2 years until the earliest of either a dementia diagnosis, death, or withdrawal from the study. The 5104 participants in the present study sample comprised those who were followed up from 1994 to 2015; 4888 participants in the sample provided their birth year. Of those, 4277 participants who provided information on their sex, age, height, educational level, and early-life socioeconomic status were included in the analysis.

Methods used for dementia diagnosis have been previously described.^19^ In brief, participants received the Cognitive Abilities Screening Instrument, a global cognition assessment (score range, 0-100 points, with lower scores indicating worse cognition),^20^ every 2 years. A score of less than 86 points prompted a standardized diagnostic evaluation, which included physical and neurological examinations and a neuropsychological test battery. Dementia diagnoses were determined at consensus conferences based on criteria from the *Diagnostic and Statistical Manual of Mental Disorders*, 4th edition.^21^ The consensus conference has been previously described.^19^

Height, an indicator of early-life nutrition^22,23^ that has been associated with dementia risk,^24,25,26^ was measured at baseline. Participants also reported their year of birth, sex, educational level, whether they had ever received antihypertensive medications or smoked, and whether they had prevalent cardiovascular disease (myocardial infarction, angina, coronary artery bypass grafting, or angioplasty), prevalent cerebrovascular disease (stroke, transient ischemic attack, or carotid endarterectomy), or prevalent diabetes. Body mass index (BMI; calculated as weight in kilograms divided by height in meters squared) was obtained,^2^ and apolipoprotein E (*APOE*) genotype was determined using published methods^27,28^ and categorized as the presence or absence of ε4 alleles.

At baseline, participants also reported information about their early-life environment, including maternal and paternal educational levels, family ability to afford basic necessities (able to meet basic needs [food, housing, clothing, and medical care] vs not able to meet ≥1 basic need) and small luxuries in childhood, family financial stability up to a participant age of 15 years (financial stability score range, 1-10, with 1 indicating least stable and 10 indicating most stable), and family household density (number of inhabitants per number of bedrooms) up to a participant age of 6 years.

### Statistical Analysis

Birth cohorts were classified as pre–World War I (1893-1913), World War I and Spanish influenza (1914-1920), pre–Great Depression (1921-1928), Great Depression (1929-1939), and World War II and postwar (1940-1949) based on historically meaningful events that were determined a priori. The pre–Great Depression group, which was the largest birth cohort, was selected as the reference group to provide statistical stability. To further describe the cohort, we created 5 categories for participant age at study entry (65-69 years, 70-74 years, 75-79 years, 80-84 years, 85-89 years, and ≥90 years). For models, we used quartiles of the distribution of participant age at study entry (65-69 years, 70-73 years, 74-79 years, and ≥80 years).

We created a combined score for parental educational levels by computing *z* scores for maternal and paternal educational levels separately, then calculating *z* scores of the sum. Self-reported childhood financial stability was divided into 4 quartiles based on score ranges (quartile 1 included stability scores of 1-3 points, quartile 2 included scores of 4-5 points, quartile 3 included scores of 6-7 points, and quartile 4 included scores of 8-10 points), with higher quartiles indicating greater stability. Height was categorized into sex-specific quartiles (for women, quartile 1 was ≤155 cm, quartile 2 was >155 cm to ≤160 cm, quartile 3 was >160 cm to ≤165 cm, and quartile 4 was >165 cm; for men, quartile 1 was ≤170 cm, quartile 2 was >170 cm to ≤175 cm, quartile 3 was >175 cm to ≤180 cm, and quartile 4 was >180 cm). Because participants in the sample reported high educational levels, we dichotomized educational levels as high school and lower vs college and higher.

We described age-specific dementia incidence rates (per 1000 person-years), overall and by birth cohort, by dividing the number of new dementia diagnoses in the age group by the number of person-years in the age group. Standard errors for log-transformed incidence rates were calculated using the delta method.^29^ We used the Fine and Gray subdistribution proportional hazards model,^30^ which provides a competing risks survival framework, with all-cause dementia as the outcome and death as a competing event. Models used baseline hazards that were stratified by quartiles of age group at study entry and time since study entry as the time axis.

To account for the potential mediation between early-life environment and dementia, we investigated the following series of models (including nested sets of covariates): (1) model 1, which included birth cohort and sex; (2) model 2, which included all variables in model 1 plus height, childhood financial stability, family ability to afford basic needs and small luxuries during childhood, and childhood household density; and (3) model 3, which included all variables in model 2 plus participant educational level. We also assessed the potential interaction between sex and birth cohort.

In sensitivity analyses, we separately included parental educational levels (for 3171 participants who provided data), *APOE* genotype (for 3554 participants who provided data), and vascular risk factors (for 4062 participants who provided data; vascular risk factors included prevalent cardiovascular disease, prevalent cerebrovascular disease, prevalent diabetes, and having ever received antihypertensive medication or smoked), and we added these factors to the model 3 variables. All analyses were conducted using R software, version 3.5.1 (R Project for Statistical Computing).

## Results

Among 5104 participants in the present study sample, 4888 participants provided their birth year. Of those, 4277 participants (the mean [SD] age, 74.5 (6.4) years; 2519 women [58.9%]) who provided information on height, educational level, and early-life socioeconomic variables, were included in the analysis. Birth cohorts comprised 483 participants in the pre–World War I cohort, 985 participants in the World War I and Spanish influenza cohort, 1491 participants in the pre–Great Depression cohort, 906 participants in the Great Depression cohort, and 412 participants in the World War II and postwar cohort.

During a total of 24 378 person-years, 730 participants developed dementia. Among the 611 participants who provided their birth year but were not included in the analysis sample, the follow-up period was shorter (median, 0 years; interquartile range [IQR], 0-5 years) compared with those in the analysis sample (median, 8 years; IQR, 4-12 years), but their characteristics were otherwise similar to participants included in the analysis. The maternal educational level in the pre–World War I birth cohort was 4 years lower (median, 8 years; IQR, 8-12 years) than that of the pre–Great Depression cohort (median, 12 years; IQR, 8-13 years) and later birth cohorts (Great Depression cohort, median, 12 years; IQR, 10-14 years; World War II and postwar cohort, median, 12 years; IQR, 12-15 years) (Table 1). The paternal educational level was 8 years (IQR, 7-13 years) for the pre–World War I birth cohort, 10 years (IQR, 8-13 years) for the pre–Great Depression birth cohort, and 12 years (IQR, 8-15 years and 11-16 years, respectively) for the Great Depression cohort and the World War II and postwar cohort.

**Table 1.** Selected Participant Characteristics by Birth Cohort^a^

Characteristic	No. (%)				
	Pre-WWI (1893-1913)	WWI and Spanish influenza (1914-1920)	Pre–Great Depression (1921-1928)	Great Depression (1929-1939)	WWII and postwar (1940-1949)
Total No.	483	985	1491	906	412
Age at baseline, median (IQR), y	85 (82-88)	78 (76-80)	71 (69-74)	71 (69-74)	67 (66-68)
Female sex	328 (68)	596 (61)	861 (58)	508 (56)	219 (53)
Educational level ≥ college	150 (31)	342 (35)	666 (45)	648 (72)	331 (80)
Height, median (IQR), cm					
Women	155 (150-160)	157 (155-163)	160 (155-165)	163 (157-165)	163 (158-168)
Men	172 (168-175)	172 (168-175)	175 (170-178)	175 (173-180)	175 (173-180)
Years of follow-up, median (IQR)	5 (3-9)	8 (5-12)	11 (6-16)	7 (4-10)	4 (2-4)
Vascular risk factors at baseline^b^					
BMI, median (IQR)	26 (23-28)	26 (24-29)	27 (24-31)	27 (25-31)	27 (24-30)
Systolic blood pressure, median (IQR)	144 (130-161)	142 (129-158)	137 (125-151)	133 (121-146)	134 (122-147)
Ever used hypertension medication	201 (44)	426 (45)	605 (42)	371 (45)	173 (45)
Ever smoked	196 (43)	468 (49)	809 (56)	452 (54)	183 (47)
Diabetes	24 (5)	99 (10)	169 (12)	85 (10)	48 (12)
Heart disease	114 (25)	197 (21)	270 (19)	109 (13)	32 (8)
Cerebrovascular disease	59 (13)	115 (12)	128 (9)	67 (8)	11 (3)
*APOE* genotype^c^	87 (20)	221 (25)	336 (25)	212 (28)	99 (29)
Childhood socioeconomic factors					
Parental years of education, median (IQR)^d^					
Mother	8 (8-12)	10 (8-12)	12 (8-13)	12 (10-14)	12 (12-15)
Father	8 (7-13)	8 (8-13)	10 (8-13)	12 (8-15)	12 (11-16)
Ability to afford basic needs and small luxuries					
Neither	44 (9)	113 (11)	174 (12)	48 (5)	13 (3)
Basic needs only	134 (28)	311 (32)	476 (32)	186 (21)	42 (10)
Both	305 (63)	561 (57)	841 (56)	672 (74)	357 (87)
Financial stability^e^					
Quartile 1	101 (21)	265 (27)	404 (30)	140 (15)	50 (12)
Quartile 2	97 (20)	224 (23)	321 (21)	234 (26)	87 (21)
Quartile 3	141 (29)	263 (27)	437 (29)	260 (29)	97 (24)
Quartile 4	144 (30)	233 (23)	293 (20)	272 (30)	178 (43)
Household density, median (IQR)^f^	2 (1-2)	2 (1-2)	2 (1-2)	2 (1-2)	2 (1-2)

Abbreviations: BMI, body mass index (calculated as weight in kilograms divided by height in meters squared); IQR, interquartile range; WWI, World War I; WWII, World War II.

^a^ Includes 4277 participants with at least 1 follow-up visit who provided information on birth year, age, sex, height, educational level, ability to afford basic needs and small luxuries during childhood, childhood financial stability, and childhood household density.

^b^ Includes only the 4065 participants who provided information on baseline vascular risk factors (BMI, systolic blood pressure, use of hypertension medications, and smoking, diabetes, heart disease, and cerebrovascular disease status).

^c^ Includes only the 3720 participants who provided information on *APOE* genotype.

^d^ Includes only the 3511 participants who provided information on maternal educational level and the 3629 participants who provided information on paternal educational level.

^e^ Quartile 1 indicates least financial stability and quartile 4 indicates most financial stability.

^f^ Measured as number of inhabitants per number of bedrooms.

A total of 305 of 483 participants (63%) in the pre–World War I birth cohort reported that their families were able to afford basic needs and small luxuries during childhood compared with 841 of 1491 participants (56%) in the pre–Great Depression birth cohort. The proportion of those able to afford basic needs and small luxuries was greater in more recent birth cohorts (672 of 906 participants [74%] in the Great Depression cohort and 357 of 412 participants [87%] in the World War II and postwar cohort). A similar pattern was observed with regard to childhood financial stability across birth cohorts, with the proportion of participants in the most stable financial quartile increasing from 144 of 483 participants (30%) in the pre–World War I cohort to 178 of 412 participants (43%) in the World War II and postwar cohort.

A comparison of earliest (pre-World War I) to most recent (World War II and postwar) birth cohorts indicated that median height increased by 8 cm for women (from 155 cm [IQR, 150-160 cm] to 163 cm [IQR, 158-168 cm]) and 2 cm for men (from 173 cm [IQR, 168-175 cm] to 175 cm [IQR, 173-180 cm]). Childhood household density was identical across birth cohorts, at 2 inhabitants per bedroom (IQR, 1-2 inhabitants). In the pre–World War I birth cohort, 150 of 483 participants (31%) completed college compared with 331 of 412 participants (80%) in the World War II and postwar birth cohort, with the largest difference in educational level between the pre–Great Depression cohort (666 of 1491 participants [45%] completed college) and the Great Depression cohort (648 of 906 participants [72%] completed college). Smoking was more prevalent among the pre–Great Depression cohort (809 of 1491 participants [56%] had ever smoked) and the Great Depression cohort (452 of 906 participants [54%] had ever smoked), while heart disease (114 of 483 participants [25%] in the pre–World War I cohort vs 32 of 412 participants [8%] in the World War II and postwar cohort) and cerebrovascular disease (59 of 483 participants [13%] in the pre–World War I cohort vs 11 of 412 participants [3%] in the World War II and postwar cohort) were less prevalent in the more recently born cohorts.

Unadjusted dementia incidence increased with age for all birth cohorts (Figure; eTable 1 in the Supplement). Age-specific dementia incidence was similar for the pre–World War I, World War I and Spanish influenza, and pre–Great Depression cohorts and was lower in the Great Depression cohort, with the exception of participants aged 75 to 79 years. The World War II and postwar cohort had a similar dementia incidence for participants aged 65 to 69 years and a higher incidence for participants aged 70 to 74 years compared with those in the Great Depression cohort.

**Figure.**
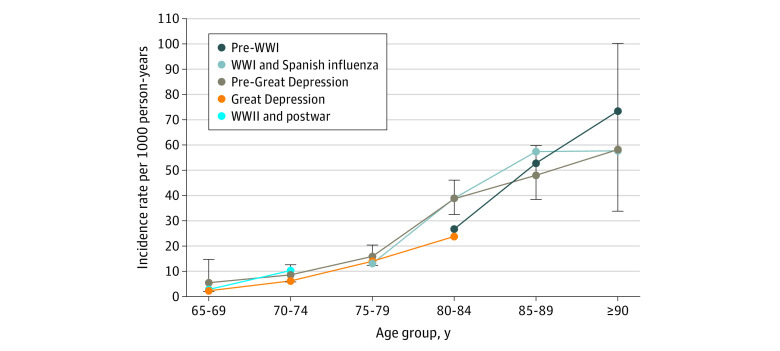
Age-Specific Dementia Incidence by Birth Cohort The graph includes participants with information available on birth cohort, age, sex, height, childhood financial stability, ability to afford basic needs during childhood, childhood household density, participants’ educational level, and parental educational levels. Age-specific dementia incidence rates were calculated by dividing the number of new dementia diagnoses in the age group by the number of person-years in the age group. Error bars indicate 95% CIs, which are shown for the pre–Great Depression birth cohort (n = 3174) as an example. WWI indicates World War I; WWII, World War II.

After adjusting for age and sex, dementia risk was lower for the Great Depression cohort (hazard ratio [HR], 0.67; 95% CI, 0.53-0.85) (model 1 in Table 2) and remained lower for the World War II and postwar cohort (HR, 0.62; 95% CI, 0.29-1.31) compared with the referent pre–Great Depression birth cohort. The 2 shortest height quartiles were associated with higher dementia risk (quartile 1, HR, 1.71; 95% CI, 1.35-2.17; quartile 2, HR, 1.43; 95% CI, 1.12-1.83) compared with the tallest height quartile (quartile 4; referent group) (model 2 in Table 2). In model 2, participants in quartiles with the least childhood financial stability had a lower risk of dementia (quartile 1, HR, 0.73; 95% CI, 0.59-0.90; quartile 2, HR, 0.81; 95% CI, 0.67-0.98) compared with those in the quartile with the most financial stability (quartile 4; referent group). With regard to childhood household density, an increase of 1 inhabitant per bedroom was associated with an 8% decrease in dementia risk (HR, 0.92; 95% CI, 0.85-0.99). The association between more recent birth cohort and risk of dementia persisted after accounting for early-life socioeconomic variables (model 2 in Table 2).

**Table 2.** Association of Birth Cohort and Socioeconomic Status in Early Life With Incident Dementia^a^

Characteristic	Hazard ratio (95% CI)		
	Model 1^b^	Model 2^c^	Model 3^d^
Birth cohort			
Pre-WWI	0.91 (0.71-1.17)	0.89 (0.69-1.14)	0.86 (0.67-1.11)
WWI and Spanish influenza	1.08 (0.90-1.31)	1.08 (0.89-1.31)	1.05 (0.87-1.27)
Pre–Great Depression	1 [Reference]	1 [Reference]	1 [Reference]
Great Depression	0.67 (0.53-0.85)	0.68 (0.54-0.87)	0.71 (0.56-0.90)
WWII and postwar	0.62 (0.29-1.31)	0.65 (0.31-1.37)	0.69 (0.32-1.45)
Female sex	1.13 (1.00-1.29)	1.14 (1.01-1.30)	1.12 (0.98-1.27)
Height^e^			
Quartile 1	NA	1.71 (1.35-2.17)	1.68 (1.32-2.13)
Quartile 2	NA	1.43 (1.12-1.83)	1.42 (1.11-1.82)
Quartile 3	NA	1.61 (1.26-2.07)	1.60 (1.25-2.06)
Quartile 4	1 [Reference]	1 [Reference]	1 [Reference]
Childhood financial stability^f^			
Quartile 1	1 [Reference]	0.73 (0.59-0.90)	0.71 (0.58-0.88)
Quartile 2	NA	0.81 (0.67-0.98)	0.80 (0.67-0.97)
Quartile 3	NA	0.96 (0.81-1.13)	0.94 (0.80-1.11)
Quartile 4	NA	1 [Reference]	1 [Reference]
Ability to afford basic needs and small luxuries			
Neither	NA	1.20 (0.94-1.52)	1.17 (0.92-1.49)
Basic needs only	NA	1.17 (1.00-1.37)	1.15 (0.99-1.34)
Both	1 [Reference]	1 [Reference]	1 [Reference]
Higher childhood household density	NA	0.92 (0.85-0.99)	0.91 (0.84-0.98)
Participant educational level ≤ high school	NA	NA	1.23 (1.07-1.40)

Abbreviations: NA, not applicable; WWI, World War I; WWII, World War II.

^a^ Includes 4277 total participants.

^b^ Model 1 includes birth cohort, age, and sex.

^c^ Model 2 includes birth cohort, age, sex, height, childhood financial stability, childhood ability to afford basic needs, and childhood household density.

^d^ Model 3 includes birth cohort, age, sex, height, childhood financial stability, childhood ability to afford basic needs, childhood household density, and participant educational level.

^e^ Quartile 1 indicates shortest height, and quartile 4 indicates tallest height.

^f^ Quartile 1 indicates least financial stability and quartile 4 indicates most financial stability.

In model 3, which accounted for participant educational level, the associations of birth cohort and early-life socioeconomic status with dementia risk remained similar, with dementia risk lower for the Great Depression (HR, 0.71; 95% CI, 0.56-0.90) and World War II and postwar (HR, 0.69; 95% CI, 0.32-1.45) cohorts compared with the referent pre–Great Depression cohort and lower in quartiles with the least financial stability (quartile 1, HR, 0.71; 95% CI, 0.58-0.88; quartile 2, HR, 0.80; 0.67-0.97) compared with the most financial stability in childhood (model 3 in Table 2). The association between birth cohort and dementia incidence did not differ by sex (eTable 2 in the Supplement). In sensitivity analyses, the results were similar when parental educational level, *APOE* genotype, and vascular risk factors were individually included (eTable 3 in the Supplement).

## Discussion

Among those born at the turn of the 20th century through the mid-20th century who participated in the ACT study, the age-specific dementia incidence was lower for participants born in 1929 and later compared with those born earlier. This trend was not explained by recalled childhood socioeconomic status and measured height, which reflect early-life environment, nor was it explained by educational level and vascular risk as an older adult. The literature on secular dementia trends reports a decrease in dementia incidence starting in the 1990s.^1,2,3,4,5^ This timing is consistent with participants in the 1929 to 1939 birth cohorts who are entering the eighth decade of life, when dementia risk increases.^2,4,31^ Political and economic changes during the first half of the 20th century may have had different implications for dementia risk based on the participant’s age during those experiences.^32^ Analysis by birth cohort captures this intersection of age and calendar time. Our results suggest that societal-level changes in the first half of the 20th century that were not captured by the individual early-life measures or the educational levels used in this study may have been associated with decreases in dementia incidence.

The 40% decrease in the US mortality rate from 1900 to 1940 was likely owing to the decrease in infectious diseases,^33^ which disproportionately occur in the young. The decrease in dementia incidence observed in the ACT study began with birth cohorts who were born in the middle of this period. These early-life health gains may be factors in the decreased dementia incidence. Although we accounted for family-level socioeconomic status variables and height, these variables may not have captured all changes, such as economic innovation^13^ and nutritional improvement,^12^ that may have been associated with decreases in mortality. In addition, variables included in this study may not have captured public heath improvements during this period.^33^ It is possible that unmeasured differences were more important for assessing dementia risk by birth cohort than the socioeconomic factors we measured.

Across birth cohorts, participants with lower financial status and greater household density in childhood had a lower risk of developing dementia, which is inconsistent with our hypothesis and the results of previous studies.^34,35^ While the Great Depression was a time of financial hardship, those in the pre–Great Depression and the World War I and Spanish influenza birth cohorts were the least likely to report the ability to afford both basic needs and small luxuries, and they had the smallest proportion of participants reporting the most stable childhood financial quartile. This pattern may reflect problems with measurement or sample selection. Participant responses may reflect experiences in later childhood and early adolescence, as recall of early-life experiences may be difficult. In contrast, parental educational levels, which were constant throughout childhood and adolescence for most of the birth cohorts, were higher for the World War I and Spanish influenza cohort and the pre–Great Depression cohort compared with cohorts born earlier. This pattern suggests a higher early-life standard of living in the more recent birth cohorts. Another possibility is that because these 2 birth cohorts were the oldest, those who survived to participate in the study were able to compensate for adverse early-life environments or had less accurate recall than younger participants.

Our study considered death as a competing risk, while a previous case-control study did not.^34,35^ Most ACT participants were members of Kaiser Permanente Washington (formerly Group Health) when they were younger than 65 years, during which they primarily received health insurance through large employers. It is likely that those with lower financial status and higher household density during childhood survived adverse experiences to be able to participate the sample.

Together with height, an individual’s parental educational level, childhood financial stability, and childhood household density are likely to reflect their early-life environment. These variables did not explain the decrease in dementia incidence among the more recent birth cohorts. In a minimally adjusted model, the decrease in dementia incidence began with the Great Depression birth cohort, suggesting that societal-level experiences during later childhood to adolescence may have been more important than those during the in-utero through early childhood phase. If this earliest stage of life were important for dementia incidence, we would expect those born in the Great Depression cohort to have the greatest dementia risk. The largest difference in college completion was found between the pre–Great Depression and Great Depression birth cohorts. This disruption to economic opportunity for those born in the pre–Great Depression years may have had implications for dementia risk. The inclusion of late-life vascular risk factors did not appreciably alter the association between a more recent birth cohort and a lower incidence of dementia, which is consistent with analyses of the Einstein Aging Cohort^10^ and the Framingham Heart Study, which considered the cohort of study entry.^1^

We found similar associations between birth cohort and decreased dementia incidence in 2 previous studies. An analysis of the English Longitudinal Study of Aging examined 2 birth cohorts based on birth-year median (1902-1925 and 1926-1943),^9^ and an analysis of the Einstein Aging Study used a data-focused approach to detect a changing point in continuous birth years.^10^ Our birth cohort categories were based on historically meaningful events. Because the ACT study is larger than the Einstein Aging Study, we were able to separate participants born after 1928 into 2 groups. In the ACT study, the most recent birth cohort (1940–1949) had higher educational levels and childhood financial stability compared with cohorts born earlier. Such categorization also allowed for the separation of worldwide economic disruption from family-level financial stability.

Our analysis may not have captured differences in adult social experiences. Educational level is associated with subsequent occupation and employment patterns. However, birth cohort may reflect experience of events during the 20th century that had broad implications, regardless of educational level. For example, men born in the first 2 decades of the 20th century are likely to have served in the armed forces during World War II and to have benefitted from the GI bill. Men and women from those birth cohorts would also have benefitted from the postwar economic expansion. Our analysis did not capture such adult experiences.

### Limitations

Our study has several limitations. Participants in older cohorts necessarily had to survive longer to be included in the study. Because the greatest risk factor for dementia is age, the requirement of survival among the pre–World War I and World War I and Spanish influenza birth cohorts as a requirement to enter the ACT study may create differences in dementia risk that are difficult to detect in these groups. Our results suggest that the most recent birth cohorts may continue to experience lower age-specific dementia incidence. However, follow-up period is shorter in these birth cohorts. The ACT study participants are from 1 health system in the Pacific Northwest, and their educational level is high. The cohort is a random sample of age-eligible members of Kaiser Permanente Washington; results therefore reflect this specific population but may not be generalizable to the US population. Our results are consistent with a sample from the Bronx, New York,^10^ and a nationally representative sample from the United Kingdom,^9^ suggesting that the decrease in dementia incidence by birth cohort may be a widespread phenomenon. Because ACT study participants may be socioeconomically advantaged, the measures of early-life environment included in this study may not be sensitive enough to detect meaningful differences that have implications for dementia incidence trends by birth cohort.

The study did not include key health variables from later in the life course that are associated with dementia risk, notably midlife hypertension, hearing loss, late-life depression, diabetes, physical inactivity, and social isolation.^6^ As the ACT study is currently collecting data on most of these variables, future studies will be able to more fully capture life-course dementia risk factors. As a long-standing study, the follow-up included substantial age overlap of multiple birth cohorts, which had been a limitation in previous studies.^9^ Dementia diagnosis procedures have been consistent throughout the study. The large size of the ACT study and the theoretical basis of the cohort groups allowed for the inclusion of 2 cohort groups born after 1928 that aligned with historically meaningful events, whereas previous studies have considered only 1 group born after the mid-1920s.^9,10^

Dementia incidence has decreased in more recent birth cohorts. Our measures of early-life socioeconomic status and educational level do not account for these differences in this study population. Birth cohort may reflect other historical and social changes that occurred during childhood or adulthood.

## Conclusions

The historical perspective of dementia risk highlights the need to consider social risk factors across the life course. Further follow-up is required to confirm dementia incidence patterns of those born during World War II and postwar and of those with the oldest ages who were born during the Great Depression period, which may illuminate differences by early-life environment not yet evident.

## Notes

Supplement. **eTable 1.** Age-Specific Dementia Incidence by Birth Cohort **eTable 2.** Association of Birth Cohort With Time to Incident Dementia, Stratified by Sex **eTable 3.** Association of Birth Cohort, Early-Life Socioeconomic Status, Parental Education, *APOE* Status, and Vascular Risk Factors With Incident Dementia
