# Supplementary material for: Association of Demographic and Early-Life Socioeconomic Factors by Birth Cohort With Dementia Incidence Among US Adults Born Between 1893 and 1949
Source: JAMA Netw Open. 2020 Jul 27;3(7):e2011094. doi: 10.1001/jamanetworkopen.2020.11094 (PMC8794045; doi:10.1001/jamanetworkopen.2020.11094)
Supplement: Supplement. — eTable 1. Age-Specific Dementia Incidence by Birth Cohort eTable 2. Association of Birth Cohort With Time to Incident Dementia, Stratified by Sex eTable 3. Association of Birth Cohort, Early-Life Socioeconomic Status, Parental Education, APOE Status, and Vascular Risk Factors With Incident Dementia [file jamanetwopen-e2011094-s001.pdf]

Supplementary Online Content

Tom SE, Phadke M, Hubbard RA, Crane PK, Stern Y, Larson EB. Association of demographic and early-life socioeconomic factors by birth cohort with dementia incidence among US adults born between 1893 and 1949. *JAMA Netw Open.* 2020;3(7):e2011094. doi:10.1001/jamanetworkopen.2020.11094

eTable 1. Age-Specific Dementia Incidence by Birth Cohort

eTable 2. Association of Birth Cohort With Time to Incident Dementia, Stratified by Sex

eTable 3. Association of Birth Cohort, Early-Life Socioeconomic Status, Parental Education, *APOE* Status, and Vascular Risk Factors With Incident Dementia

This supplementary material has been provided by the authors to give readers additional information about their work.

© 2020 Tom SE et al. *JAMA Network Open*. All rights reserved.

eTable 1. Age-Specific Dementia Incidence by Birth Cohort[a]

Age group	No. of person-years	No. of cases	Rate per 1000 person-years (95% CI)[b]
**65-69 years**			
All	1868	7	3.7 (1.8, 7.9)
Pre-WWI	--	--	--
WWI and Spanish influenza	--	--	--
Pre-Great Depression	724	4	5.5 (2.1, 14.7)
Great Depression	431	1	2.3 (0.3, 16.5)
WWII and postwar	713	2	2.8 (0.7, 11.2)
**70-74 years**			
All	5648	44	7.8 (5.8, 10.5)
Pre-WWI	--	--	--
WWI and Spanish influenza	49	0	--
Pre-Great Depression	3030	26	8.6 (5.8, 12.6)
Great Depression	2083	13	6.2 (3.6, 10.7)
WWII and postwar	487	5	10.3 (4.3, 24.7)
**75-79 years**			
All	7156	106	14.8 (12.2, 17.9)
Pre-WWI	--	--	--
WWI and Spanish influenza	1295	17	13.1 (8.2, 21.1)
Pre-Great Depression	3780	60	15.9 (12.3, 20.4)
Great Depression	2078	29	14.0 (9.7, 20.1)
WWII and postwar	3	0	--
**80-84 years**			
All	6564	239	36.4 (32.1, 41.3)
Pre-WWI	300	8	26.7 (13.4, 53.4)
WWI and Spanish influenza	2238	87	38.9 (31.5, 48.0)
Pre-Great Depression	3229	125	38.7 (32.5, 46.1)
Great Depression	798	19	23.8 (15.2, 37.4)
WWII and postwar	--	--	--
**85-89 years**			
All	4035	212	52.5 (45.9, 60.1)
Pre-WWI	739	39	52.8 (38.6, 72.3)
WWI and Spanish influenza	1638	94	57.4 (46.9, 70.2)
Pre-Great Depression	1646	79	48.0 (38.5, 59.8)

© 2020 Tom SE et al. *JAMA Network Open*. All rights reserved.

Great Depression	12	0	--
WWII and postwar	--	--	--
**≥90 years**			
All	1883	122	64.8 (54.2, 77.4)
Pre-WWI	845	62	73.4 (57.2, 94.1)
WWI and Spanish influenza	814	47	57.7 (43.4, 76.8)
Pre-Great Depression	224	13	58.2 (33.8, 100.2)
Great Depression	--	--	--
WWII and postwar	--	--	--

[a]n = 3174. 95% confidence intervals shown for Pre-Great Depression birth cohort as an example; sample with information on birth cohort, age, sex, height, childhood financial stability, ability to afford needs, childhood household crowding, education, and parental education.

[b]Delta method to calculate standard errors.

© 2020 Tom SE et al. *JAMA Network Open*. All rights reserved.

eTable 2. Association of Birth Cohort With Time to Incident Dementia, Stratified by Sex

Birth Cohort	Women (n = 2512) HR (95% CI)	Men (n = 1765) HR (95% CI)	*P* value[a]
Pre-WWI	1.00 (0.73, 1.38)	0.70 (0.46, 1.07)	.41
WWI and Spanish influenza	1.19 (0.93, 1.53)	0.91 (0.67, 1.23)	.17
Pre-Great Depression	Reference	Reference	NA
Great Depression	0.75 (0.54, 1.03)	0.67 (0.46, 0.97)	.98
WWII and postwar	0.87 (0.33, 2.26)	0.49 (0.15, 1.63)	.59

[a]*P* value from the interaction of sex and birth cohort in a pooled-sex model that includes birth cohort, age, sex, height, childhood financial stability, ability to afford needs, childhood household crowding, and education.

© 2020 Tom SE et al. *JAMA Network Open.* All rights reserved.

eTable 3. Association of Birth Cohort, Early-Life Socioeconomic Status, Parental Education, *APOE* Status, and Vascular Risk Factors With Incident Dementia

**Characteristic**	**Model 1 (n = 3174) HR (95% CI)**	**Model 2 (n = 3720) HR (95% CI)**	**Model 3 (n = 4065) HR (95% CI)**
**Birth cohort**			
Pre-WWI	1.00 (0.74, 1.36)	0.96 (0.73, 1.26)	0.92 (0.70, 1.20)
WWI and Spanish Flu	1.07 (0.85, 1.34)	1.06 (0.86, 1.31)	1.04 (0.85, 1.27)
Pre-Depression	Reference	Reference	Reference
Great Depression	0.67 (0.50, 0.88)	0.71 (0.54, 0.92)	0.70 (0.54, 0.90)
WWII - later	0.76 (0.34, 1.72)	0.56 (0.22, 1.43)	0.61 (0.28, 1.36)
**Woman**	1.16 (1.00, 1.36)	1.11 (0.97, 1.27)	1.09 (0.95, 1.26)
**Height**			
Quartile 1 (shortest)	1.81 (1.37, 2.40)	1.68 (1.28, 2.14)	1.70 (1.33, 2.18)
Quartile 2	1.40 (1.05, 1.87)	1.43 (1.10, 1.86)	1.41 (1.10, 1.81)
Quartile 3	1.64 (1.22, 2.19)	1.58 (1.21, 2.07)	1.60 (1.24, 2.07)
Quartile 4 (tallest)	Reference	Reference	Reference
**Childhood family finances**			
Quartile 1 (least stable)	0.76 (0.59, 0.98)	0.73 (0.58, 0.91)	0.70 (0.57, 0.87)
Quartile 2	0.77 (0.61, 0.96)	0.82 (0.67, 1.00)	0.79 (0.65, 0.96)
Quartile 3	0.93 (0.76, 1.13)	0.96 (0.80, 1.15)	0.92 (0.77, 1.09)
Quartile 4 (most stable)	Reference	Reference	Reference
**Ability to afford needs**			
Neither basics nor small luxuries	1.19 (0.88, 1.61)	1.19 (0.92, 1.55)	1.17 (0.91, 1.50)
Basics only	1.10 (0.91, 1.32)	1.20 (1.02, 1.41)	1.14 (0.98, 1.34)
Basics and small luxuries	Reference	Reference	Reference
**Childhood household crowding (increasing)**	0.91 (0.83, 1.00)	0.89 (0.82, 0.97)	0.92 (0.85, 1.00)
**Up to high school (vs college+)**	0.86 (0.73, 1.00)	1.20 (1.04, 1.39)	1.23 (1.07, 1.41)
**Parental education**			
Q1	1.05 (0.84, 1.32)		
Q2	1.19 (0.95, 1.50)		
Q3	1.03 (0.82, 1.30)		
Q4	Reference		
***APOE* (any vs none)**		1.69 (1.47, 1.95)	
**Baseline vascular risk factors**			
BMI			0.99 (0.98, 1.01)
Systolic blood pressure			1.00 (1.00, 1.00)
Ever use of hypertension medication			0.96 (0.83, 1.10)
Ever smoked			0.89 (0.78, 1.01)

© 2020 Tom SE et al. *JAMA Network Open*. All rights reserved.

Diabetes			1.11 (0.90, 1.37)
Heart disease			0.97 (0.82, 1.15)
Cerebrovascular disease			1.02 (0.82, 1.26)

Model 1 includes birth cohort, age, sex, height, childhood financial stability, ability to afford needs, childhood household crowding, education, and parental education.

Model 2 includes birth cohort, age, sex, height, childhood financial stability, ability to afford needs, childhood household crowding, education, and *APOE* genotype status.

Model 3 includes birth cohort, age, sex, height, childhood financial stability, ability to afford needs, childhood household crowding, education, and vascular risk factors.

© 2020 Tom SE et al. *JAMA Network Open*. All rights reserved.
